# Arcuate kisspeptin neurons display two types of calcium events: Regulation by sex, gonads, and senktide

**DOI:** 10.1111/jne.70298

**Published:** 2026-10-27

**Authors:** R. Anthony DeFazio, Christian R. Burgess, Laura L. Burger, Amanda G. Gibson, Suzanne M. Moenter

**Affiliations:** ^1^ Department of Molecular and Integrative Physiology University of Michigan Ann Arbor Michigan USA; ^2^ Michigan Neuroscience Institute University of Michigan Ann Arbor Michigan USA; ^3^ Department of Psychology and Neuroscience Regis University Denver Colorado USA; ^4^ Department of Internal Medicine University of Michigan Ann Arbor Michigan USA; ^5^ Department of Obstetrics and Gynecology University of Michigan Ann Arbor Michigan USA; ^6^ The Reproductive Sciences Program University of Michigan Ann Arbor Michigan USA

**Keywords:** arcuate, calcium imaging, GnRH, pulse generator, reproduction

## Abstract

The pattern of gonadotropin‐releasing hormone (GnRH) release controls reproduction in vertebrates and is regulated by feedback from the gonads. Evidence is accumulating that steroid‐sensitive hypothalamic arcuate kisspeptin–neurokinin B–dynorphin (KNDy) neurons drive GnRH pulses. KNDy neurons exhibit coordinated, large amplitude calcium signals in vivo that precede LH pulses; coordinated events persist in brain slice recordings of these cells. We used cre‐lox to target GCaMP6f expression to KNDy neurons. Brain slice calcium imaging was done to identify putative unitary calcium events. Newly devised analyses identified two types of calcium events (fast and slow) in intact and gonadectomized male and female adult mice. Neurokinin B and the neurokinin‐3 receptor agonist senktide are strong activators of action potential firing in KNDy neurons. The effect of senktide on calcium events over 5‐min baseline and treatment periods was determined. Gonadectomy unexpectedly increased GCaMP6f fluorescence; illumination levels were adjusted to standardize initial levels. Senktide increased the frequency of fast and slow events; this effect was enhanced in gonadectomized mice for fast events. Senktide increased fast event amplitude only in gonadectomized mice. For slow events, in contrast, senktide increased amplitude in all groups, with the increase being larger in gonadectomized than intact mice. Differential regulation by gonadectomy and senktide modulation of fast and slow events suggests two separate mechanisms for these events, which likely also differ from the large amplitude events observed in vivo in KNDy neurons.

## 1 INTRODUCTION

Gonadotropin‐releasing hormone (GnRH)‐producing neurons form the final common central pathway controlling reproduction in vertebrates. GnRH stimulates synthesis^1^ and secretion of the gonadotropins luteinizing hormone (LH) and follicle‐stimulating hormone (FSH) by the anterior pituitary. The gonadotropins activate gonadal functions, including steroidogenesis, and steroids feed back to regulate GnRH release. GnRH is secreted in an episodic manner in males^2^ and through most of the reproductive cycle in females.^3^ This episodic release is essential for pituitary function,^4^ and changes in pulse frequency are further required to help drive differential release of LH and FSH during the female reproductive cycle.^5^ Central volleys of multiunit neural activity measured in the primate infundibular/arcuate region are correlated with pulses of LH release,^6^ and the unidentified neurons associated with this episodic signal were termed the “GnRH pulse generator.” The nature of the pulse generator and how steroid feedback changes frequency are two long‐standing questions. Steroid receptors other than estrogen‐receptor beta are difficult to detect in GnRH neurons,^7^, ^8^, ^9^, ^10^ suggesting that steroid feedback signals are likely processed in afferent neurons. Several papers point to arcuate hypothalamic neurons, termed KNDy neurons, that express kisspeptin, neurokinin B, and dynorphin as well as steroid receptors as a strong candidate for both steroid feedback and pulse generation.^11^, ^12^, ^13^, ^14^, ^15^, ^16^

The neurophysiologic mechanisms underlying the pulse generator remain under investigation. Calcium imaging in neurons has long been used as a surrogate for action potential firing.^17^, ^18^ Strong depolarization during the action potential opens voltage‐gated calcium channels resulting in calcium influx that can be detected with fluorescent indicators.^19^ Calcium imaging can also detect increased intracellular calcium arising from other sources including influx via N‐methyl‐D‐aspartate (NMDA) receptors,^20^ release from mitochondria^21^ and thapsigargin‐induced endoplasmic reticulum stress.^22^ In vivo fiber photometry studies of KNDy neurons expressing the genetically‐encoded calcium indicator GCaMP6s identified population‐level calcium events likely reflecting increases in intracellular calcium in multiple cells.^13^ These were termed synchronized events (SEs) and preceded LH pulses. In vivo imaging at the single‐cell level revealed potential roles for individual KNDy neurons in initiation and termination of such events across multiple cells.^23^, ^24^ In acutely prepared brain slices, synchronized calcium events (miniature SEs) in KNDy neurons persisted and were associated with bursts of action potentials.^13^, ^25^

GCaMP6s used in the above in vivo and in vitro studies has a good signal‐to‐noise ratio, but it has slower kinetics than GCaMP6f; this can limit the resolution of calcium events derived from single action potentials.^26^ Here, we used GCaMP6f combined with a higher sampling rate. We tested if calcium events were dependent upon sex, gonadal status, and/or neuromodulator action.

## 2 METHODS

*Animals*. Tg(Kiss1‐cre)J2‐4Cfe/J (JAX 023426) mice were crossed with B6;129S‐*Gt(ROSA)26Sor* ^*tm95.1(CAG‐GCaMP6f)Hze*^/J (JAX 024105) mice to produce offspring heterozygous for both alleles for imaging studies. These mice, along with Tac2‐cre^27^ (a gift from Dr. Martin Myers) and Tg(Gnrh1‐cre)1Dlc/J (JAX 021207) mice crossed with B6;129S‐*Gt(ROSA)26Sor* ^*tm95.1(CAG‐GCaMP6f)Hze*^/J were used for tissue harvest for western blots. Mice had ad libitum access to Envigo 2916 chow and water and were maintained at 21°C–23°C on a 14L:10D light cycle with lights on at 0300 eastern standard time. The Institutional Animal Care and Use Committee of the University of Michigan approved all procedures.

*Experimental design*. Adult mice 67–190 days old were ovariectomized (OVX), orchidectomized (ORX), or sham operated under isoflurane anesthesia with bupivacaine as a local analgesic 5–19 days before study. In sham‐operated females, estrous cycles were monitored by vaginal cytology for at least 7 days before recording, and mice were studied on diestrus. A few animals were OVX and treated with a Silastic capsule to replace estradiol (OVX + E, western blot analysis of GCaMP6f only). Number of mice, days post‐surgery, body, uterine, and seminal vesicle mass at the time of brain slice preparation are shown in Table 1, as are the number of slices and regions of interest (ROIs) analyzed. Calcium imaging was performed on acutely prepared brain slices. A baseline recording period was followed by bath‐applied treatment with senktide (100 nM, Phoenix Pharmaceuticals, Burlingame, CA), a synthetic peptide agonist of neurokinin 3 receptors. At least four mice per group were studied, and no more than three slices per mouse were used (Table 1). The number of slices was used as the experimental unit (N), and calcium events were analyzed at the ROI level as described below. All event analyses were done blind to the experimental groups.

**TABLE 1:** Mean ± SEM of mouse biometrics and numbers of ROIs analyzed under different conditions.

Animals	Parameter	Diestrus	OVX	Male	ORX
	# Mice	4	6	4	4
	# Slices	6	8	5	10
	Age (days)	104.8 ± 9.0	144.8 ± 34.1	110.5 ± 30.4	115.3 ± 52.4
	Days post‐surgery	11.8 ± 4.9	8.3 ± 3.1	9.0 ± 6.7	8.0 ± 0.8
	Body mass (g)	22.4 ± 1.9	23.5 ± 2.4	31.0 ± 2.6	26.8 ± 5.6
	Uterine mass (mg)	61.7 ± 15.0	36.6 ± 8.8		
	Seminal vesicle mass (mg)			337.5 ± 57.9	106.5 ± 77.5
Raw ∆*F*/*F*0	# Total ROIs	185	273	153	339
	# ROIs with fast events during baseline	137	87	76	104
	# ROIs with slow events during baseline	158	76	90	105
	# ROIs with fast events during senktide	172	273	134	305
	# ROIs with slow events during senktide	180	273	152	305
Penumbra‐subtracted	# Total ROIs	181	270	146	329
	# ROIs with fast events during baseline	81	69	53	65
	# ROIs with slow events during baseline	105	60	66	76
	# ROIs with fast events during senktide	132	256	77	280
	# ROIs with slow events during senktide	148	265	115	292

Abbreviations: ORX, orchidectomized; OVX, ovariectomized; ROIs, regions of interest.

*Brain slice preparation*. All extracellular solutions were bubbled with 95% O_2_/5% CO_2_ throughout experiments and kept at 30°C–31°C with an inline‐heating unit (Warner Instruments). Coronal brain slices (300 μm) containing the hypothalamic arcuate nucleus were prepared with a VT1200S (Leica Biosystems) from 0730 to 0830 EST; recordings were obtained from 0830 to 1430 EST. The brain was rapidly removed and placed in ice‐cold sucrose saline solution containing (in mM): 250 sucrose, 3.5 KCl, 26 NaHCO_3_, 10 d‐glucose, 1.25 Na_2_HPO_4_, 1.2 MgSO_4_, and 3.8 MgCl_2_ (350 mOsm). Slices were incubated for 30 min at room temperature (~21°C–23°C) in 50% sucrose saline and 50% artificial cerebrospinal fluid (ACSF, containing [in mM]: 135 NaCl, 3.5 KCl, 26 NaHCO_3_, 10 d‐glucose, 1.25 Na_2_HPO_4_, 1.2 MgSO_4_, 2.5 CaCl_2_, 315 mOsm, pH 7.4). In most animals, three slices included the arcuate region, one of which typically had relatively few cells. The middle slice was studied first, then the caudal slice was examined and if ≥10 cells were clearly visible, it was studied. If not, the rostral slice was examined and studied. In a few animals, all three slices had sufficient cells for study. No differences were observed in analysis response based on region. Slices were then transferred to 100% ACSF solution at room temperature for 0.5–6 h before recording. To increase stability over long recordings, slices were attached to poly‐l‐lysine coated coverslips (Corning BioCoat, Discovery Labware, Bedford, MA) and allowed to adhere for 10s before being transferred to the recording chamber and secured with an anchor.

*Western blot analysis*. Tissue punches from the preoptic area (GnRH‐cre driver) or arcuate nucleus (Tac2 or Kiss1 cre driver) were homogenized in radioimmunoprecipitation assay buffer with protease and phosphatase inhibitors (Halt; Thermo Scientific), and protein concentrations were determined by bicinchonic acid assay. Lysates (15 μg) were resolved on 4%–15% acrylamide gels (Bio‐Rad) and transferred onto PVDF (BioRad). Blots were probed with goat anti‐green fluorescent protein (GFP) (Novus NB100‐1770; 1:5000) which binds to GCaMP6f; donkey anti‐goat horse‐radish perxoxidase (HRP) secondary antibody was used for detection (Invitrogen A15999; 1:8000). The blot was stripped and reprobed with rabbit anti‐GAPDH (Abcam ab9485; 1:1000) with goat anti‐rabbit HRP secondary antibody (Abcam ab6721; 1:8000) as a loading control. Immunoreactivity was detected with SuperSignal West Dura and quantified by densitometry on the iBright Imaging System (Invitrogen).

*Calcium imaging parameters*. The GCaMP6f fluorescence was monitored using an ORCA‐Fusion Gen‐III sCMOS camera (Hamamatsu, Japan) mounted to the upright BX51WI microscope with a 20× objective (Olympus XLUMPLFLN20XW). Epifluorescent illumination was provided by a X‐Cite TURBO LED system (Excelitas, Pittsburgh, PA). A standard GFP filter cube was used: excitation: 470/40 nm; emission: 525/50 nm; band pass beam splitter: 495 nm (Chroma U‐M41017). CellSens Dimension software (Evident Scientific, Waltham, MA) was used to define imaging experiments and acquire images. Timing was controlled using the Olympus U‐RTC real‐time controller (Evident Scientific). Images were acquired at 10 Hz using an exposure time of 20 ms. Binning (4 × 4) was used to increase sensitivity, resulting in an image pixel size of 1.3 × 1.3 μm. To reduce the size of data files, images were cropped to the minimum size necessary to capture GCaMP6f cells the arcuate. GCaMP6f fluorescence levels were consistently lower in gonad‐intact mice of both sexes. The illumination intensity was adjusted to achieve a similar baseline fluorescence histogram.

*Calcium imaging analysis*: The output of cellSens acquisition software is a three‐dimensional array (two spatial axes, *X* and *Y*, and time, *t*). Each time point in the array is an image of the *XY* plane and will be referred to as a frame. The time of each frame is preserved throughout the analysis. Arrays were imported to MATLAB using the BioFormats plugin.

Slice movement occurs over time, thus motion correction in the *XY*‐plane was performed using the NoRMCorre algorithm^28^ as implemented in the MATLAB version of CaImAn.^29^ The minimum pixel value of each frame was subtracted and then each frame was normalized by the maximum pixel value of the frame, followed by a Gaussian spatial filter. To confirm effective motion correction, the alignment of cells at the beginning, middle, and end of the experiment were compared by dividing the array into thirds based on time and taking the maximum value for each pixel to form an image. The three resulting images were merged into an RGB image (red for the first third, green for the second, and blue for the third). Good alignment resulted in white cells in this RGB image. In most cases, rigid correction (translation and rotation in the *XY*‐plane) was sufficient. If rigid correction failed, subsequent non‐rigid motion correction (translation, rotation, and stretch/compression in the *XY*‐plane) was done using the NoRMCorre algorithm. After successful motion correction was confirmed, the alignment was applied to the original array before further analysis.

ROI selection was performed in a manner to reduce bias based on initial activity level or baseline fluorescence. For each pixel over time, the mean, maximum, and standard deviation for both the raw fluorescence (*F*) and the ∆*F*/*F*0 were obtained and made into a rendition. All six renditions were simultaneously visible for ROI selection. Cells (ROIs) had to have a coherent shape and be comprised of ~5–10 adjacent pixels. Cells that were relatively dim but active exhibit higher standard deviation, allowing their inclusion even though mean signal was similar to the background of the image. Maximum fluorescence intensity was useful for identifying cells that were largely stable over time but had a few larger events that might be lost in the mean projection. Mean intensity was typically less noisy, thus useful for adding confidence to ROI selection based on the other criteria. ∆*F*/*F*0 calculations produced signals normalized to the baseline fluorescence, allowing cells that were active later in the recording to have roughly equal amplitude calcium signals compared to those early in the imaging session before bleaching.

Upon selection of a cell, a custom algorithm estimated the full‐width‐at‐half‐maximum (FWHM) for the ROI spatial profile at 45° increments in the *XY*‐plane, resulting in eight‐sided ROIs. An annular penumbra was created by drawing a second eight‐sided object 4 points out from the original pixels (5.2 μm) to create the inner boundary and an additional 2 points (2.6 μM) to create the outer boundary of the penumbra. The penumbra sometimes included signal that is arguably associated with the cell presumed to underlie an ROI (such as from a neuronal process); however, the majority of the penumbra lacked evident signal. The mean intensity of the pixels contained in each ROI and penumbra were calculated for each frame of the stack and stored as a function of time. The mean of the penumbra was multiplied by 0.75 before it was subtracted from the ROI mean to avoid negative intensity values for ROIs with baseline fluorescence near background.

To calculate ∆*F*/*F*0, *F*0 was estimated using a modification of the method of Konnerth.^30^ This consisted of smoothing (boxcar average over 1 s) and a backwards search over the trace for the next minimum. Because some of the events of interest are long (>30s), the backwards search was conducted over the entire trace, and the baseline was estimated as a slope between the search start and the next backwards minimum. *F*0 and ∆*F*/*F*0 were stored for both the ROI and the penumbra‐subtracted (pensub) ROI.

*Event detection*. Calcium events were detected in two ways: peak detection based on a rising phase followed by a falling phase (custom algorithm termed “Custard”), and Custard peak detection after heavy smoothing to remove fast transient events and identify the set of slow events.

Custard is based on the Cluster algorithm used for analysis of time‐series hormone values.^31^ In Custard, a test period consisting of a user‐identified number of samples (0.5 s window, five samples) is compared with the previous period of the same length using Student's *t*‐test. If the test is significant, the sample is labeled as “up” or “down” depending on the direction of change. One or more ups followed by one or more downs is considered a possible event; the minimum event is one up followed by a down. Baseline ends at the first “up” of an event and is the mean of the previous five samples. Events are further subject to amplitude and width thresholds. Peak is the local maximum of the event. Amplitude is the peak minus the preceding baseline. Width is estimated by looking forwards and backwards from the peak for the time at which the calcium signal crosses the peak minus 50% of the amplitude. The minimum amplitude for inclusion as an event was based on the sigmaest noise estimation function from the MLspike MATLAB package. The algorithm was run for each ROI for both raw and penumbra‐subtracted data. This value was multiplied by 2, 3, 4, or 5 to generate a set of amplitude threshold values. The results for all four thresholds were evaluated by eye, with the number of false positives decreasing and the number of false negatives increasing with the multiplier. A threshold factor of 4× sigmaest value was selected as a balance between false positives and negatives; statistical significance of identified events was similar for 3× and 5×. The minimum half width was set to 0.3 s based on the time course of the unitary calcium signal under ideal conditions.^26^

To detect slow events evident in the data series, the Custard algorithm (1 s window, 10 samples) was run again after heavily filtering the signal to remove fast calcium events. We used a zero‐phase 0.2 Hz low‐pass digital filter to remove fast changes in the calcium signal. Custard event detection was done on the resulting smoothed trace as described above to identify slow events. Detected slow events were removed from the total number of identified events when the peak of a slow event was within ±2 s of a previously identified event, leaving only fast events. Each slow event could remove one event (the closest event to the peak of the slow event) from the total number of identified events. Slow events were also detected that were not part of the original identified events.

*Data analysis approach*. Custard analysis measured amplitude and full width half maximum as an estimate of decay time of each detected event across an entire recording. Analysis results were separated into baseline and treatment periods; events during the first 2 min after a solution change were not included in quantification. This allowed for complete bath turnover between treatments. Frequency was calculated as the number of events divided by duration for each ROI, whereas amplitude and width were the median of events within that ROI over the test period. The mean ± SEM of these parameters was calculated for each treatment period at the slice level and then group mean ± SEM was calculated. ROIs with zero events during both baseline and treatment were removed from all analyses unless events were detected during a subsequent 20 mM K+ treatment (tested in 20 of 29 slices). To visualize the proportion of ROIs responding to senktide treatment, the fractional response to senktide (mean treatment ∆*F*/*F*0 divided by ∆*F*/*F*0 mean baseline) was determined for each ROI in each animal model. This assessment is independent of detection method.

*Statistical analysis*. Statistical comparison of *F*0 after illumination adjustment (Figure 1) was done with two‐way ANOVA in GraphPad Prism 11. Statistical analysis of calcium event data was conducted in R; statistical packages used included rstatix,^32^ lme4,^33^ lmerTest,^34^ afex,^35^ emmeans.^36^ Plots were made with the packages ggplot2^37^ and cowplot.^38^ Tables were made with the flextable package.^39^ Additional packages used for data wrangling, file management, and miscellaneous tasks included tidyverse,^40^ pracma,^41^ fastmatch,^42^ here,^43^ openxlsx,^44^ officer,^45^ performance,^46^ and lattice.^47^ Plot styling and rendering used viridis,^48^ scales,^49^ Cairo,^50^ extrafont,^51^ ggpubr,^52^ and ggbeeswarm.^53^

**FIGURE 1:**
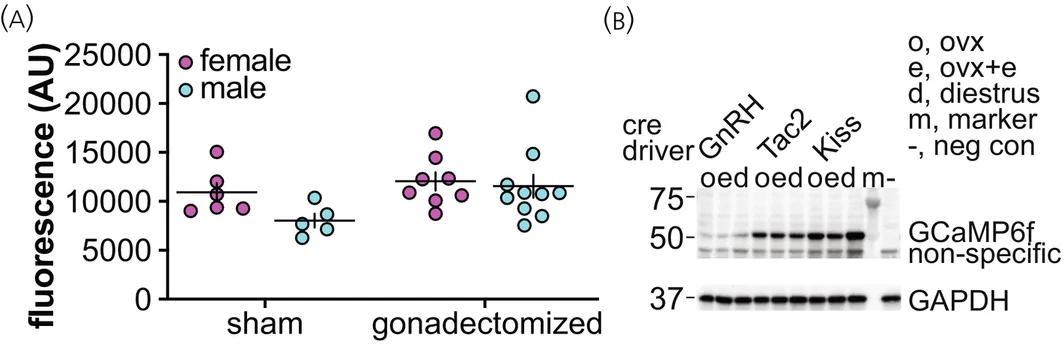
(A) Baseline fluorescence after adjustment of illumination intensity. Each dot represents the mean fluorescence of the ROIs in a slice (*n* = 6 slices diestrus, *n* = 5 male, *n* = 8 OVX, *n* = 10 ORX). Slices from females had higher adjusted baseline fluorescence than males (*p* = .0492), whereas there was no difference in adjusted baseline fluorescence by gonad status (*p* = .1450) or interaction between sex and gonadectomy (*p* = .2794, two‐way ANOVA). (B) Western blot showing bands for GCaMP6f and GAPDH. The non‐specific band on the top blot is typical for the NOVUS GFP antibody used. Ovariectomized plus estradiol (ovx + e) treatment was examined in this screen but not utilized in further experiments. ORX, orchidectomized; OVX, ovariectomized; ROIs, regions of interest.

Linear mixed models (LMMs) were used to analyze ROI‐level data. LMMs can account for the dependencies among data attributable to experimental design (e.g., different numbers of ROI/slice, different numbers of slices per mouse) and permit missing data.^54^ The general model used was outcome ~sex * gonad status * treatment + (1|slice) + (1|mouse). To compare event detection with raw ∆*F*/*F*0 versus penumbra subtraction, the model used was outcome ~ analysis + (1|slice) + (1|mouse). For frequency, the analysis was conducted on the square root transformation of frequency, as 0 is meaningful; data are plotted on a pseudolog scale. For amplitude and width, a log_10_ transformation of the measure was conducted. Type III tests with effects coding were used, as recommended by Singmann and Kellen.^54^ The Kenward–Roger approximation was used for estimation of degrees of freedom for linear mixed models. For some measures, the model fit was flagged as singular with both the slice and mouse included as random effects variables. Given the biological relationship of ROIs that come from the same slice or animal, however, these random factors were left in the model. Post hoc comparisons were made using pairwise tests of estimated marginal means (emmeans). If the three‐way interaction between sex, gonad status, and senktide treatment was significant, post hoc analysis was conducted on this interaction. If the three‐way interaction was not significant, then the two‐way interactions were considered, and post hoc analyses were conducted for all significant two‐way interactions. For multiple pairwise comparisons, *p* values were adjusted using Holm's method. Reported estimates for post hoc comparisons were back‐transformed to the response value for ease of interpretation.

To compare the distribution of ROI‐level responses to senktide across all four experimental groups, the cumulative frequencies of the fold‐change of mean ∆*F*/*F*0 after senktide (treatment / baseline) were assessed with the Anderson–Darling test using the kSamples package in R.^55^ When this omnibus test was significant, post hoc Anderson–Darling tests were conducted to compare the distributions from (1) diestrous and male, (2) OVX and ORX, (3) diestrous and OVX, and (4) male and ORX groups, and *p* values were adjusted for multiple comparisons using Holm's method.

*Code accessibility*. The detection and analysis code used is freely available online at https://github.com/syncytium2/KNDy-Ca-1. The R analysis code is freely available online at https://github.com/gibson-amandag/ArcKissCa_analysis. Analyses were conducted on a Dell Precision 5860 Tower with Intel Xeon w7‐2495X 24‐core processor. Statistical analyses were conducted on a MacBook Pro with Apple M3 Pro Chip.

*AI statement*. All image processing, event analysis, and statistics code was generated by the investigators or taken from public/published repositories as indicated. Claude Code (Anthropic), an AI coding assistant (version 2.1.133, Claude Sonnet 4.6 model; May 2026), was used by R.A.D. to help write the R plotting routines that produce Figures 6 and S1; the figures were generated by running this code on the authors' data. Claude Opus 4.7 (Anthropic, via claude.ai; 2026) was used to plan and organize this coding work. Figure 7: Claude Code (Claude Opus 5 model; version 2.1.266; August–September 2026) was used to compute the per‐ROI fold change in mean ∆*F*/*F*0 plotted in Figure 7. R.A.D. directed AI‐assisted coding, reviewed the code before use, and checked the resulting figures against the underlying data. The AI tools with access to the code base were used under individual accounts with the setting that permits training on user content turned off. The data are from mice (procedures approved by the University of Michigan IACUC) and contain no human‐subject or personally identifiable information.

## 3 RESULTS

*Gonadectomy affects GCaMP6f fluorescence*. At the outset of these studies, a difference was noted in the fluorescent signal from intact versus gonadectomized mice. Using 100% illumination intensity, the peak of the baseline fluorescence histogram for intact mice was between 10% and 20% of the dynamic range of the camera, whereas the fluorescence histogram from gonadectomized mice of both sexes extended further into the camera's range. To achieve a similar baseline fluorescence histogram as observed in intact mice, illumination intensity was adjusted so that the 90th percentile of the pixel fluorescence intensity histogram was below 20,000 to accommodate fluorescence increases during treatment without camera saturation (maximum intensity value 65,535). As a consequence, the illumination intensity was 5%–30% for slices from gonadectomized mice and 90%–100% for intact mice. Failure to make this adjustment would preclude testing senktide as signals from gonadectomized animals could saturate during treatment. Baseline fluorescent levels of ROIs after standardization are shown in Figure 1A. This difference in uncorrected intensity was not attributable to different levels of GCaMP6f within the sensitivity of western blot analysis (Figure 1B). GnRH neurons are rare compared to KNDy neurons, likely accounting for less GCaMP6f in samples from the GnRH cre driver.

*Two data streams were generated and are analyzed throughout the results*. These studies were performed with standard (i.e., not multiphoton) imaging. A low‐power image of the medial basal hypothalamus showing with GCaMP6f signal in the arcuate region is in Figure 2A. To test for possible interference from out‐of‐focus light, we compared data from raw and penumbra subtracted (pensub) traces. Figure 2B–D shows how the penumbra of a ROI was defined and subtracted. Note there is extension of some ROI‐associated signal into the upper penumbra region in this example, however the vast majority of this and other ROIs' penumbra regions lacked evident signal.

**FIGURE 2:**
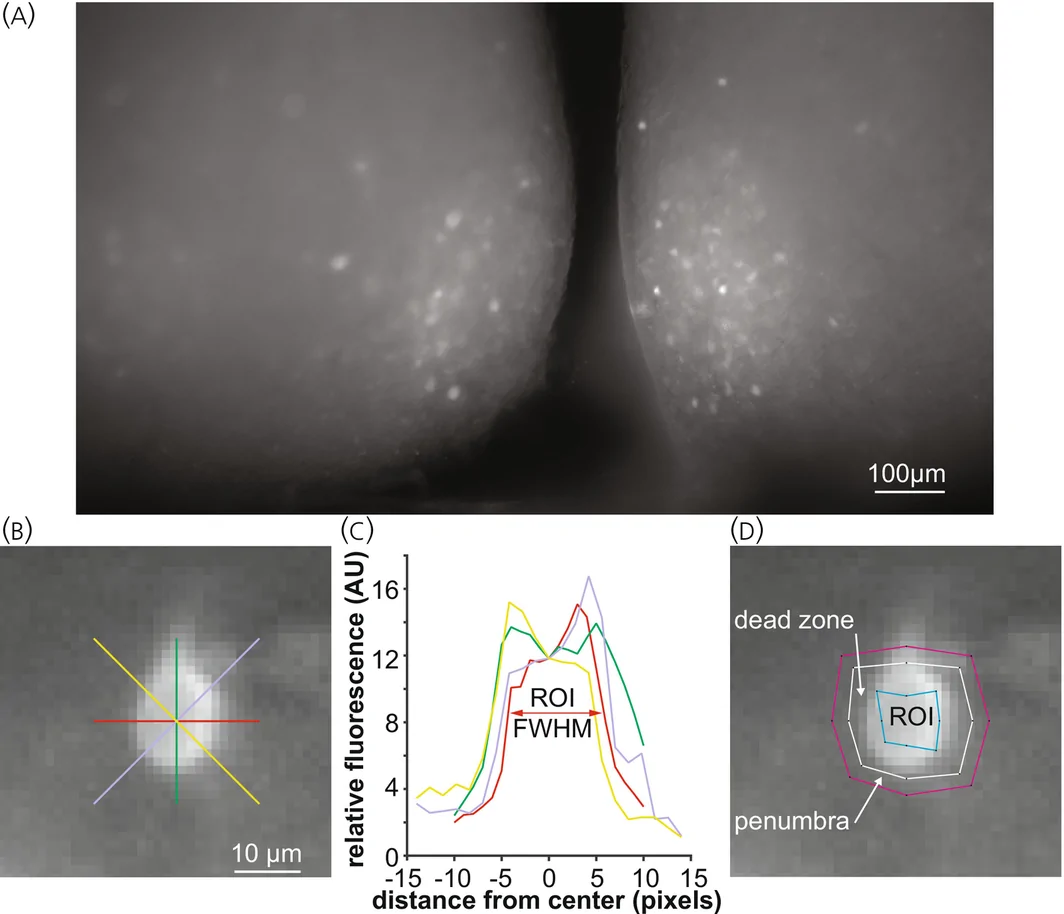
Generation of custom ROI and penumbra. (A) Low‐power image of GCaMP6f fluorescence in the arcuate region. (B) image of a cell with 45° increments in the *XY*‐plane used for penumbra subtraction illustrated. (C) relative fluorescence along lines on the left image, colors correspond. The calculation of the horizontal limb of the ROI by FWHM of the red line is illustrated. (D) ROI (within cyan), dead zone (between cyan and white) and penumbra (between white and magenta) are shown. FWHM, full width at half maximum; ROIs, regions of interest.

Both raw and pensub traces were converted to ∆*F*/*F*0 for event detection (Figure 3). Pensub traces were noisier because of subtraction but had higher amplitude ∆*F*/*F*0 because of smaller *F*0 (note *Y*‐axis change from Figure 3A to Figure 3B for fluorescence intensity).

**FIGURE 3:**
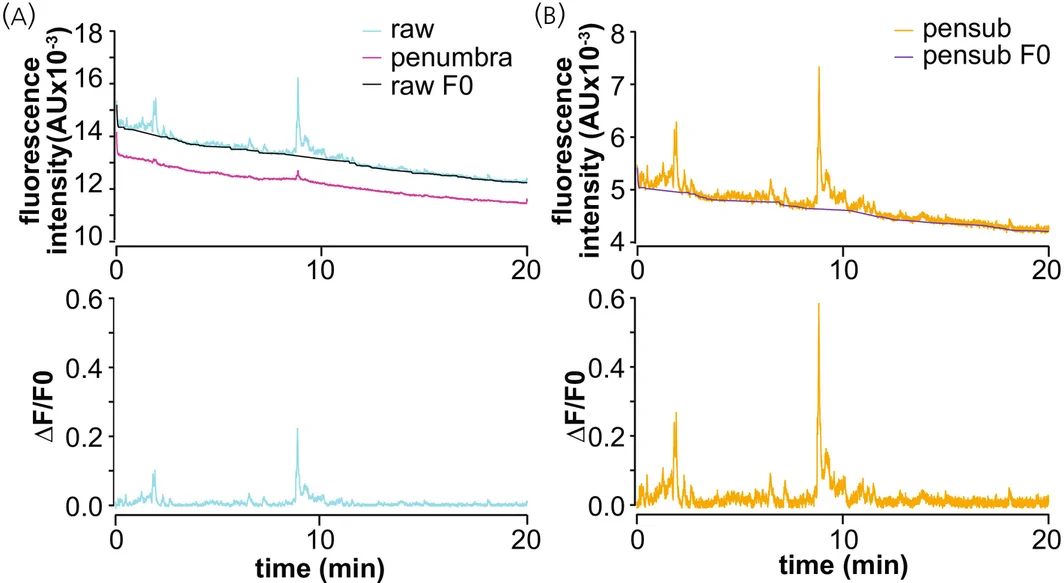
Processing of calcium imaging data. (A) Top, traces of raw data, penumbra‐subtracted data and raw *F*0; bottom, ∆*F*/*F*0 of raw data. (B) Top, penumbra subtracted (pensub) trace and pensub *F*0; bottom, ∆*F*/*F*0 of pensub data.

*KNDy neurons exhibit calcium events with two different timecourses*. Event detection was run on raw and pensub ∆*F*/*F*0 traces. Detected events from the raw ∆*F*/*F*0 trace are shown in cyan squares in Figure 4A; events detected during the period of bath exchange (yellow shading in Figure 4) were not included in statistical analyses. Visual examination of detected events revealed some resembled classic unitary calcium events thought to be associated with single action potentials, termed “fast,” and more prolonged events with more sustained deviations from baseline, termed “slow.” To separate these events, raw ∆*F*/*F*0 traces were filtered to remove fast transients, and event detection was again applied to the filtered trace (Figure 4B). Final analysis is shown in Figure 4C, where grey boxes indicate the events in A that were removed because of slow events; cyan boxes with black × are the finalized fast events. Figure 4D is the same plot as Figure 4C but utilizes penumbra‐subtracted data; note the change in *Y*‐axis scale. This analysis resulted in the loss of some events that were detected when the raw ∆*F*/*F*0 was analyzed (vertical cyan and magenta lines at the top of Figure 4D).

**FIGURE 4:**
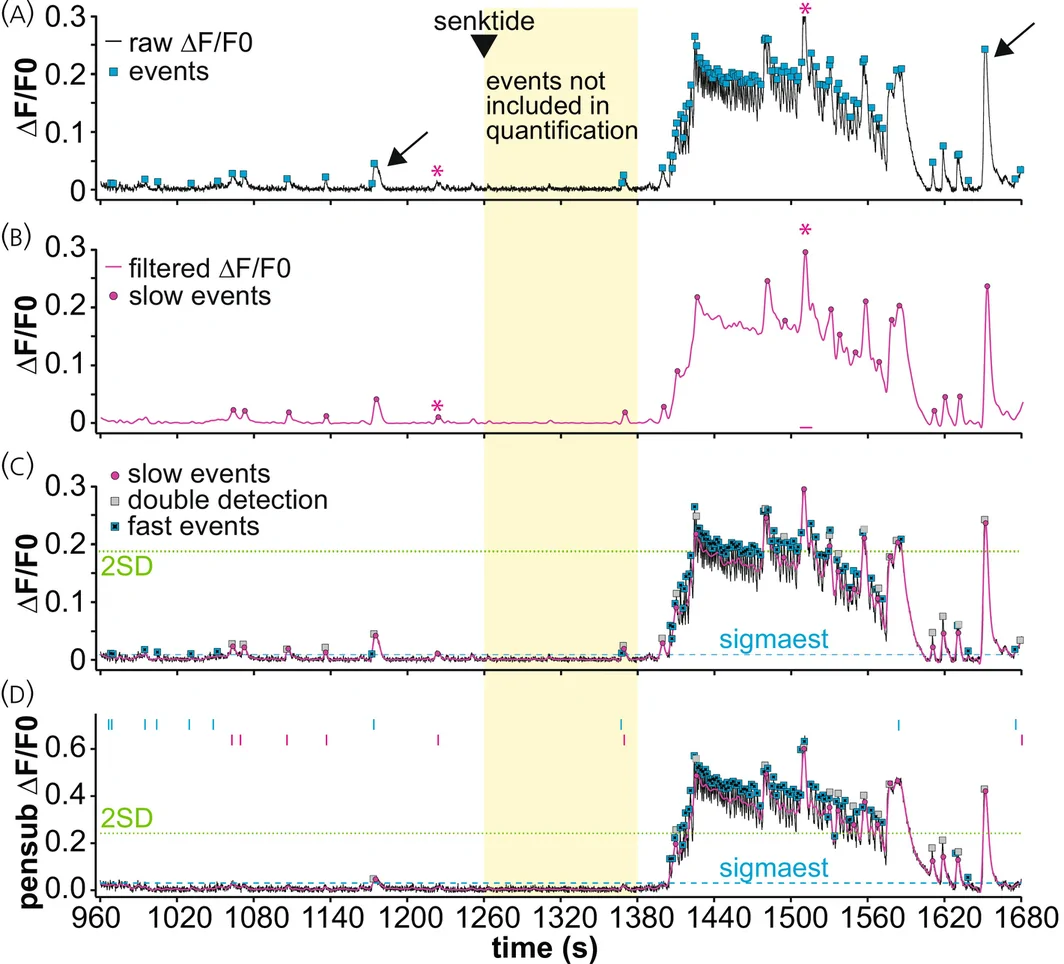
Detection of calcium events. (A) Five‐minute raw ∆*F*/*F*0 trace with representative event frequency, cyan squares show all events detected. Yellow shading shows region of bath exchange during which detected events were not included in analyses and the arrowhead shows the start of the senktide treatment. Arrows point to examples of detected events with longer durations, magenta asterisk marks two events not detected in raw data that were added by the slow event detection in (B). (B) The ∆*F*/*F*0 trace was filtered to remove events with fast dynamics. Magenta circles show events detected on the filtered trace, which were designated as slow. (C) superimposition of raw and filtered traces. Grey squares show events detected on the raw trace that occurred within 2 s of a slow event and were removed from the list of fast events (cyan squares with black ×). Dashed cyan line shows the 4× sigmaest noise threshold used in the present study; green dashed line shows 2SD used in several other reports.^13^ (D) Same presentation as C using penumbra subtracted (pensub) ∆*F*/*F*0. Vertical lines at the top of (D) show where pensub analysis missed fast (cyan) or slow (magenta) events; missed events during the senktide‐induced increase are not marked for clarity. Note *Y*‐axis scale is different in (D).

*Effects of sex, gonadal status and senktide on KNDy neuron calcium events*. The number of ROIs analyzed under different conditions is indicated in Table 1. Examples of raw ∆*F*/*F*0 traces from each group during baseline, bath exchange (yellow) and senktide treatment are shown in Figure 5. Event analysis as described above was performed to compare a 5‐min baseline to a 5‐min senktide recording using both raw ∆*F*/*F*0 and penumbra‐subtracted traces. LMM analysis of events from raw ∆*F*/*F*0 traces revealed that sex, gonadal status, and senktide treatment all interacted to alter the frequency of fast calcium events (Figure 6A left and Tables 2 and 3). Senktide increased the frequency of fast events in all groups tested. Gonadectomy magnified this increase in both sexes, but, interestingly, to a greater extent in females. In contrast to the consistent marked effects on fast event frequency, senktide only increased fast event amplitude in gonadectomized mice (Figure 6B left and Tables 2 and 3). Senktide increased the width of fast events only in gonadectomized mice. This effect is mild and not likely to be biologically relevant, more likely arising from the tightness of the data (Figure 6C left and Tables 2 and 3). For slow events, senktide increased frequency in all groups to a similar extent (Figure 6A right and Tables 4 and 5) and there was no enhancement by gonadectomy. Senktide increased slow event amplitude in both males and females (Figure 6B and Tables 4 and 5). The senktide‐induced increase in amplitude was larger in gonadectomized than intact mice. A mild effect of senktide to reduce the width of slow events was observed in males but is not likely to be of biologic significance (Figure 6C right and Tables 4 and 5). The distinct regulation of fast versus slow events by senktide argues they are biologically different.

**FIGURE 5:**
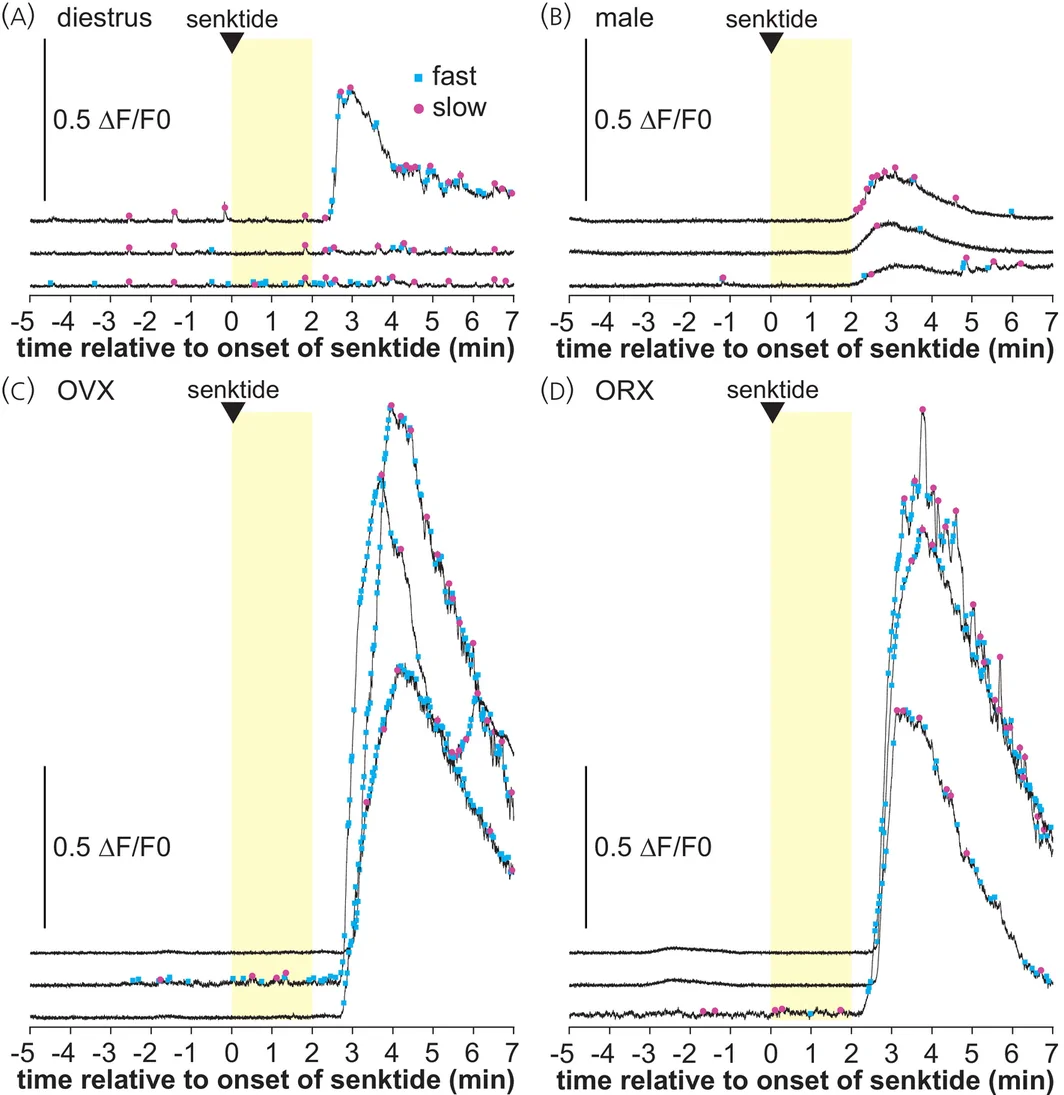
Three representative ROIs within a slice from (A) diestrous, (B) male, (C) OVX, and (D) ORX groups during baseline, bath exchange (yellow) and senktide treatment. Arrowhead indicates the start of senktide treatment. Fast events are marked with cyan squares, slow events with magenta circles. ORX, orchidectomized; OVX, ovariectomized; ROIs, regions of interest.

**FIGURE 6:**
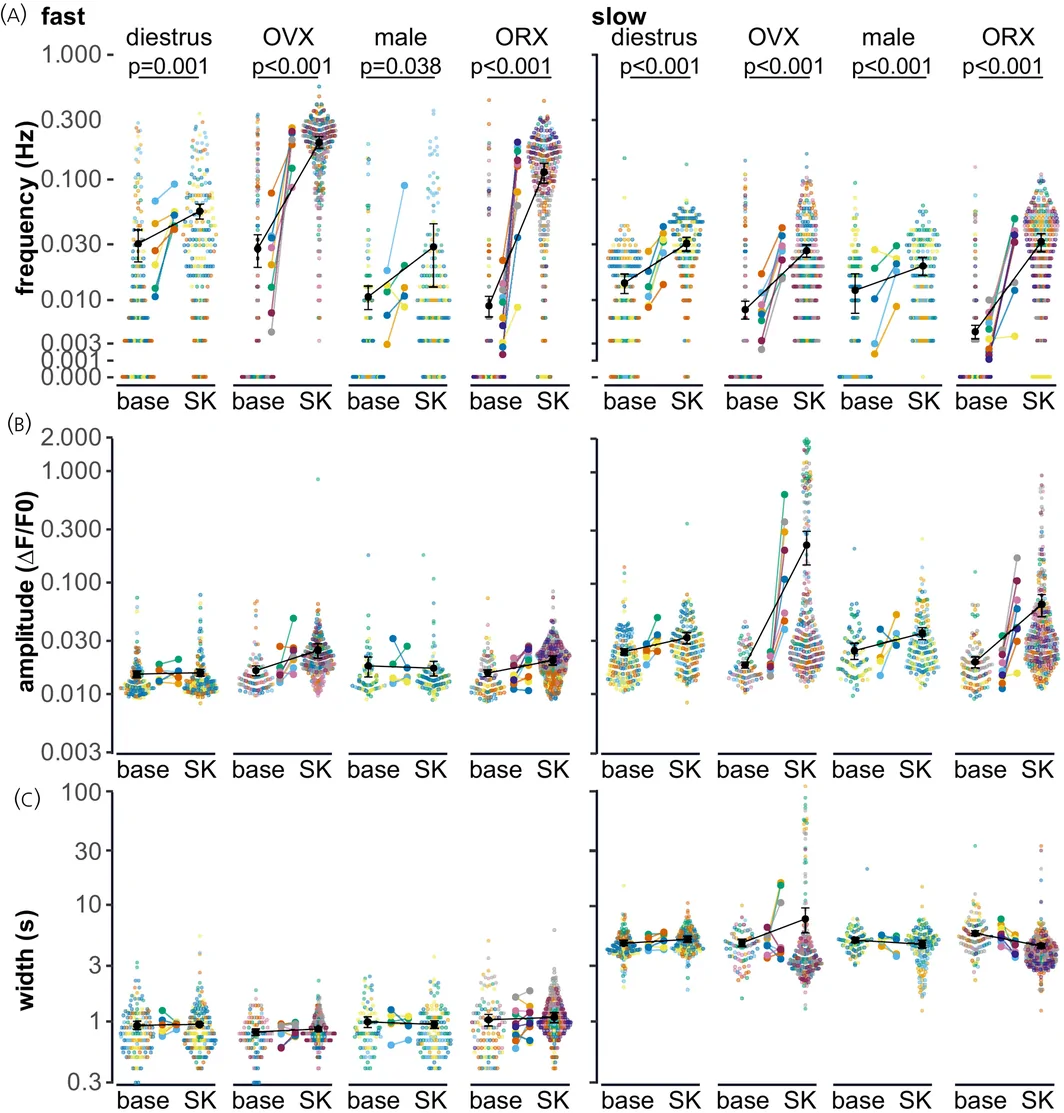
Sex, gonadal status and senktide alter calcium events in KNDy neurons, raw ∆*F*/*F*0 analysis at the ROI level. (A) Frequency of fast (left) and slow (right) events. (B) Amplitude of fast (left) and slow (right) events. (C) Width of fast (left) and slow (right) events. For frequency, small dots are # events/duration for each ROI, large dots are mean of individual ROIs in that slice (color matched) and group mean ± SEM is shown in black. For amplitude and width, small dots are the median of events in that ROI, slice and group mean data are calculated as for frequency. Pairwise comparisons of baseline versus senktide for fast and slow event frequency for each animal model shown on the graph. All other statistical results and post hoc comparisons are in Tables 2, 3, 4, 5. ROI, region of interest.

**TABLE 2:** LMM analysis of fast events detected on raw Δ *F/F* 0 data.

Variable	Frequency (Hz)			Amplitude (Δ*F*/*F*0)			Width (s)		
	*F*	df	*p*	*F*	df	*p*	*F*	df	*p*
Sex	11.00	1, 13.5	.005	0.01	1, 13.9	.928	2.47	1, 13.9	.139
Gonad	16.21	1, 13.5	.001	6.27	1, 13.9	.025	0.08	1, 13.9	.782
Senktide (SK)	1219.11	1, 1867	<.001	45.74	1, 1267	<.001	4.04	1, 1265.9	.045
Sex * gonad	0.17	1, 13.5	.690	0.48	1, 13.9	.498	1.06	1, 13.9	.321
Sex * SK	24.15	1, 1867	<.001	1.24	1, 1267	.266	0.10	1, 1265.9	.746
Gonad * SK	416.85	1, 1867	<.001	29.98	1, 1267	**<.001**	7.35	1, 1265.9	.**007**
Sex * gonad * SK	12.90	1, 1867	**<.001**	0.13	1, 1267	.719	0.73	1, 1265.9	.394

*Note*: Bolded *p*‐values indicate the highest‐order significant interactions that were further analyzed with post hoc comparisons (Table 3).

Abbreviation: LMM, linear mixed model.

**TABLE 3:** Post hoc comparison pairs for significant interactions (from Table 2) for fast events from raw ∆*F*/*F*0 analysis.

Outcome	Group level	Contrast	Estimate	95% CI	SEM	df	*t*	*p*
Frequency (sex * gonad * senktide)	Intact baseline	Female—male	0.0100	[−0.0115, 0.0315]	0.0064	16.0	1.56	.556
	GDX baseline	Female—male	0.0046	[−0.0111, 0.0203]	0.0039	7.5	1.19	.811
	Intact senktide	Female—male	0.0244	[−0.0163, 0.0652]	0.0122	16.0	2.00	.314
	**GDX senktide**	**Female—male**	0.0979	[0.0078, 0.1881]	0.0222	7.5	4.41	.**018**
	Female baseline	Intact—GDX	0.0067	[−0.0155, 0.0288]	0.0066	16.0	1.01	.811
	Male baseline	Intact—GDX	0.0013	[−0.0130, 0.0155]	0.0035	7.5	0.36	.811
	**Female senktide**	**Intact—GDX**	−0.1503	[−0.2204, −0.0802]	0.0210	16.0	**−7.16**	**<.001**
	**Male senktide**	**Intact—GDX**	−0.0767	[−0.1343, −0.0192]	0.0142	7.5	**−5.42**	.**006**
	**Intact female**	**Baseline—senktide**	−0.0282	[−0.0473, −0.0091]	0.0057	16.0	**−4.93**	.**001**
	**Intact male**	**Baseline—senktide**	−0.0138	[−0.0284, 0.0008]	0.0045	20.2	**−3.05**	.**038**
	**GDX female**	**Baseline—senktide**	−0.1851	[−0.2358, −0.1345]	0.0155	18.3	−11.96	**<.001**
	**GDX male**	**Baseline—senktide**	−0.0918	[−0.1355, −0.0481]	0.0108	7.5	−8.53	**<.001**
Amplitude (gonad * senktide)	Baseline	Intact—GDX	−0.0015	[−0.0059, 0.0029]	0.0016	16.9	−0.98	.684
	**Senktide**	**Intact—GDX**	−0.0070	[−0.0122, −0.0018]	0.0018	12.6	−3.94	.**005**
	Intact	Baseline—senktide	−0.0005	[−0.0021, 0.0011]	0.0006	16.2	−0.88	.684
	**GDX**	**Baseline—senktide**	−0.0060	[−0.0080, −0.0039]	0.0007	12.6	−8.44	**<.001**
Width (gonad * senktide)	Baseline	Intact—GDX	0.0330	[−0.1883, 0.2542]	0.0788	16.3	0.42	>.999
	Senktide	Intact—GDX	−0.0783	[−0.3071, 0.1504]	0.0782	12.2	−1.00	>.999
	Intact	Baseline—senktide	0.0141	[−0.0689, 0.0970]	0.0296	16.4	0.48	>.999
	**GDX**	**Baseline—senktide**	−0.0972	[−0.1783, −0.0161]	0.0277	12.2	−3.51	.**017**

Abbreviation: GDX, gonadectomized.

**TABLE 4:** LMM analysis of slow events detected on raw ∆*F*/*F*0 data.

Variable	Frequency (Hz)			Amplitude (Δ*F*/*F*0)			Width (s)		
	*F*	df	*p*	*F*	df	*p*	*F*	df	*p*
Sex	0.43	1, 13.7	.523	0.25	1, 14.0	.628	0.03	1, 14.2	.876
Gonad	3.15	1, 13.7	.098	0.75	1, 14.0	.402	0.05	1, 14.2	.830
Senktide (SK)	885.30	1, 1867	<.001	147.04	1, 1322	<.001	2.77	1, 1324	.096
Sex * gonad	0.48	1, 13.7	.499	1.60	1, 14.0	.226	1.03	1, 14.2	.326
Sex * SK	0.00	1, 1867	.960	5.42	1, 1322	.**020**	19.75	1, 1324	**<.001**
Gonad * SK	80.91	1, 1867	<.001	58.31	1, 1322	**<.001**	4.04	1, 1324	.**045**
Sex * gonad * SK	5.63	1, 1867	.**018**	2.44	1, 1322	.119	2.99	1, 1324	.084

*Note*: Bolded *p*‐values indicate the highest‐order significant interactions that were further analyzed with post hoc comparisons (Table 5).

**TABLE 5:** Post hoc comparison pairs for significant interactions (from Table 4) for slow events from raw ∆*F*/*F*0 analysis.

Outcome	Group level	Contrast	Estimate	95% CI	SEM	df	*t*	*p*
Frequency (sex * gonad * senktide)	Intact baseline	Female—male	0.0023	[−0.0115, 0.0162]	0.0041	15.4	0.56	>.999
	GDX baseline	Female—male	0.0005	[−0.0050, 0.0060]	0.0015	10.2	0.35	>.999
	Intact senktide	Female—male	0.0081	[−0.0151, 0.0313]	0.0069	15.4	1.18	>.999
	GDX senktide	Female—male	−0.0024	[−0.0242, 0.0194]	0.0059	10.2	−0.41	>.999
	Female baseline	Intact—GDX	0.0078	[−0.0031, 0.0187]	0.0032	15.4	2.40	.235
	Male baseline	Intact—GDX	0.0060	[−0.0048, 0.0168]	0.0029	10.2	2.04	.473
	Female senktide	Intact—GDX	0.0050	[−0.0168, 0.0269]	0.0065	15.4	0.77	>.999
	Male senktide	Intact—GDX	−0.0055	[−0.0288, 0.0178]	0.0064	10.2	−0.86	>.999
	**Intact female**	**Baseline—senktide**	−0.0183	[−0.0274, −0.0092]	0.0027	15.4	−6.75	**<.001**
	**Intact male**	**Baseline—senktide**	−0.0124	[−0.0202, −0.0047]	0.0024	17.8	−5.27	**<.001**
	**GDX female**	**Baseline—senktide**	−0.0210	[−0.0308, −0.0113]	0.0029	16.7	−7.14	**<.001**
	**GDX male**	**Baseline—senktide**	−0.0240	[−0.0368, −0.0111]	0.0035	10.2	−6.82	**<.001**
Amplitude (sex * senktide)	Baseline	Female—male	−0.0011	[−0.0102, 0.0081]	0.0033	20.3	−0.32	.751
	Senktide	Female—male	0.0078	[−0.0093, 0.0250]	0.0059	13.4	1.32	.419
	**Female**	**Baseline—senktide**	−0.0235	[−0.0323, −0.0147]	0.0031	16.8	−7.48	**<.001**
	**Male**	**Baseline—senktide**	−0.0146	[−0.0219, −0.0073]	0.0025	13.4	−5.79	**<.001**
Amplitude (gonad * senktide)	Baseline	Intact—GDX	0.0058	[−0.0037, 0.0153]	0.0035	20.2	1.68	.107
	**Senktide**	**Intact—GDX**	−0.0215	[−0.0392, −0.0038]	0.0059	11.0	−3.62	.**012**
	**Intact**	**Baseline—senktide**	−0.0065	[−0.0124, −0.0006]	0.0021	18.1	−3.04	.**014**
	**GDX**	**Baseline—senktide**	−0.0338	[−0.0447, −0.0229]	0.0036	11.0	−9.28	**<.001**

Outcome	Group level	Contrast	Estimate	95% CI	SEM	df	*t*	*p*
Width (sex * senktide)	Baseline	Female—male	−0.6406	[−1.6565, 0.3754]	0.3739	22.3	−1.71	.201
	Senktide	Female—male	0.5115	[−0.4090, 1.4319]	0.3203	13.7	1.60	.201
	Female	Baseline – senktide	−0.3573	[−0.8430, 0.1285]	0.1742	17.2	−2.05	.167
	**Male**	**Baseline—senktide**	0.7948	[0.2196, 1.3700]	0.2002	13.7	3.97	.**006**
Width (gonad * senktide)	Baseline	Intact—GDX	−0.1940	[−1.2005, 0.8124]	0.3709	22.6	−0.52	>.999
	Senktide	Intact—GDX	0.3221	[−0.6375, 1.2817]	0.3227	11.1	1.00	>.999
	Intact	Baseline—senktide	−0.0448	[−0.5525, 0.4630]	0.1837	18.6	−0.24	>.999
	GDX	Baseline—senktide	0.4714	[−0.0880, 1.0307]	0.1881	11.1	2.51	.116

To visualize if sex or gonadal status affected the percentage of ROI responding to senktide, the fractional change in mean ∆*F*/*F*0 between baseline and senktide was plotted as a cumulative distribution for each group (Figure 7). Most ROIs in all groups exhibited a robust increase in ∆*F*/*F*0 during senktide treatment, though there were differences in the distributions between groups (Anderson–Darling [AD] value = 210.16, *t* = 157.56, *p* < .001). About 10% of ROIs decreased fluorescence in gonad‐intact groups; this was largely attributable to more activity during baseline at the level of individual ROIs and was not a slice‐wide phenomenon. In contrast, ROIs from both OVX (AD = 120, *t* = 156.95, *p* < .001) and ORX (AD = 85.73, *t* = 111.71, *p* < .001) groups exhibited more robust ∆*F*/*F*0 changes during senktide than were observed in slices from sex‐matched gonad‐intact animals. In general, the magnitude of ROI‐level changes following senktide within a gonadal status category was greater in females than males (intact: AD = 2.652, *t* = 2.18, *p* = .041; gonadectomized: AD = 14.56, *t* = 17.87, *p* < .001).

**FIGURE 7:**
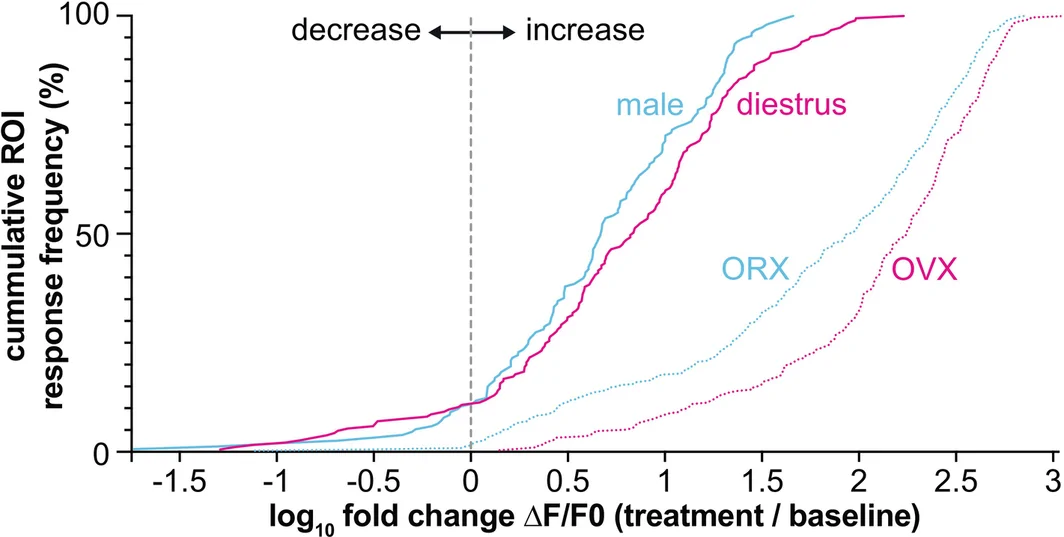
Distribution plot of the percent ROI response frequency to senktide calculated as the fractional change in fluorescence (∆*F*/*F*0) from baseline for each of the four groups. ROI, region of interest.

*Analysis of penumbra‐subtracted data confirms similar effects of sex, gonadal status*, *and senktide*. Penumbra subtraction was done as described above to ameliorate changes within an ROI stemming from out‐of‐focus light, such as that arising from fibers of passage. We first compared event parameters between these two analysis methods during the baseline recordings for each ROI (Table 6). Neither fast nor slow event width was affected by analysis approach. As expected by the smaller *F*0 denominator for the ∆*F*/*F*0 for pensub events, amplitude was higher after penumbra subtraction for both fast and slow events. Both fast and slow events were less frequent following penumbra subtraction. This most likely indicates a loss of events due to the noisier signal following penumbra subtraction. Importantly, analysis‐method‐induced changes did not alter the interpretation of either fast or slow event characteristics as the penumbra‐subtracted data largely showed the same pattern of changes as the raw ∆*F*/*F*0 (Figure S1 and Tables S1–S4). The most notable changes between analysis type were on amplitude, as expected for this transformation. This indicates the primary signals being analyzed arose from the selected ROIs and thus reflect neurobiology rather than being a result of the method of data acquisition or processing.

**TABLE 6:** ROI‐level comparison of raw vs. penumbra‐subtracted event detection analysis during baseline.

		Frequency (Hz)		Amplitude (Δ*F*/*F*0)				Width (s)		
Event type	Variable	*F*	df	*p*	*F*	df	*p*	*F*	df	*p*
Fast	Analysis	19.06	11846.8	<.001	1139.97	1652.9	<.001	3.23	1, 648.8	.073
Slow	Analysis	42.59	11846.4	<.001	781.67	1710.4	<.001	.71	1, 711.9	.400

## 4 DISCUSSION

Episodic GnRH release that varies in frequency and amplitude in a steroid‐dependent cyclic manner in females is essential for fertility. Hypothalamic arcuate kisspeptin, or KNDy neurons, have been postulated to be the generator of this episodic activity. Calcium imaging provides an approach for assessing population dynamics in these neurons, which is a necessary step to mechanistically defining the pulse‐generating mechanisms. We used this approach in brain slices and describe two types of calcium events in this cell type, both of which are sensitive to sex, gonadal status, and the KNDy neuron neuromodulator senktide.

In the past decade, calcium imaging has been used in vivo and in brain slices to study KNDy neurons. Bulk photometry first demonstrated an association between increases in fluorescence of GCaMP6s targeted to KNDy neurons that occurred in association with LH pulses.^13^ Although this approach does not allow resolution of individual cells, large repetitive increases in calcium signals associated with LH release were reasonably postulated to arise from contemporaneous activation of multiple KNDy neurons. These events have been referred to as SEs. Individual cells have been imaged using two approaches. First, gradient index lens technology was used for in vivo imaging of individual cells, testing their synchrony and whether or not leader and follower cells exist.^23^, ^24^ Two or more cells having calcium elevations in temporal proximity of each other was observed in both of these studies, providing initial corroboration of the underlying assumptions of the fiber photometry work, although there was no consensus on leader cells. Both bulk photometry and individual cellular in vivo imaging showed calcium increases in KNDy neurons preceded LH release. Second, multiple cells have been imaged in brain slices. Spontaneous calcium increases in multiple KNDy neurons persisted in this reduced preparation and were associated with bursts of action potentials.^13^, ^25^ To distinguish from the in vivo bulk photometry work, these events were called miniature SEs. Miniature SEs were shown to be acutely modified by neurotransmitters^56^, ^57^, ^58^, ^59^ as well as by differences in physiologic state.^60^, ^61^, ^62^, ^63^, ^64^

These findings support and advance the above work by broadening our understanding of the types of calcium events produced by KNDy neurons. Notably, many of the prior studies focused on large calcium events, often using a threshold of twice the SD for detection. Traditional calcium imaging analysis has often been based on the premise of a unitary calcium signal, defined as the calcium event in response to a single action potential. To embrace this approach, we employed GCaMP6f, which has a faster response and decay time than GCaMP6s^26^ and a higher sampling rate (10 vs. 2–5 Hz). We strengthened the rigor of event detection using an algorithmic noise estimate that was designed to be independent of biological increases in the calcium signal. We also employed multiple approaches for identification of ROIs to include cells with different calcium patterns and examined the effect of out‐of‐focus light in brain slices. These modifications revealed two types of calcium increases in KNDy neurons. Fast events have a rapid increase and rate of decay towards baseline similar to the GCaMP6f calcium signal produced in response to a single action potential.^26^ Slow events are less frequent, larger and have a greater width.

In addition to the technical changes in imaging and analysis approach, the present work directly compared the effects of sex, gonadal status, and the neuromodulator senktide within a single study. In the course of doing these studies, it was soon apparent that the GCaMP6f baseline fluorescence levels were altered by gonadal manipulation but apparently not by sex. This is not mentioned in prior papers examining intact versus gonadectomized mice.^62^ Initial western blot analysis indicated similar levels of GCaMP6f among animal models, suggesting this is not a result of a gonadectomized‐induced increase in expression. This observation necessitated adjustments to the illumination levels to allow fair comparison among groups and avoid saturation of the camera during senktide treatment in gonadectomized groups, but it also suggests that calcium homeostasis in KNDy neurons is modified by gonadal feedback, most likely from sex steroids.^65^ In this regard, studies have reported effects of sex or sex steroids on calcium levels. Treatment of cultured embryonic hippocampal neurons with dihydrotestosterone increased basal calcium levels.^66^ In contrast, the calcium current produced in adult GnRH neurons in brain slices in response to a single action potential is greater in orchidectomized than intact males.^67^ Sex differences were also reported in basal calcium levels in neonatal hippocampal cultures, with females being higher than males in part due to de novo synthesis of estradiol by the cultures.^66^

Sex, gonadal status, and the neurokinin B receptor agonist senktide differentially regulated the two types of calcium events. Sex and gonadal status also affected the proportion of individual ROIs responding with an increase in ∆*F*/*F*0. In the present study, senktide increased fast event frequency in all four groups, and this increase was enhanced in gonadectomized mice of both sexes compared to intact mice. This is consistent with the effects of senktide on firing rate of GFP‐identified KNDy neurons in males, which was also greater in orchidectomized than intact males, and extends this concept to females.^68^ Interestingly, the frequency of fast events during senktide was even higher in ovariectomized than orchidectomized mice. Notably, as in the prior electrophysiology work in which many cells were quiescent before senktide,^68^, ^69^, ^70^, ^71^ several ROIs were quiescent before treatment. As with fast events, slow event frequency was increased by senktide in all four animal models. Amplitude of fast events was mildly increased by senktide but only in gonadectomized mice, whereas the effect on amplitude in slow events was more pronounced and was observed in all four animal models. These latter observations, in conjunction with the minor differences in width, are consistent with the postulate that fast events resemble unitary calcium events, while slow events arise from multiple mechanisms.

Senktide is an agonist of the neurokinin 3 receptor and engages Gq‐coupled receptors that utilize increased intracellular calcium as one signaling mechanism. Its effects on calcium events are thus not surprising, but the present findings demonstrate new aspects of the calcium biology of KNDy neurons and pose several questions for future work. First, how do the types of events detected in the present work correspond to the SE correlated with LH pulses and mSE reported in brain slices? While most bulk photometry studies have focused on larger events, smaller events were noted in some studies.^24^ Figure 4 has a horizontal dashed line illustrating the 2SD threshold used for mSE identification. The vast majority of both fast and slow events observed in the present work would not be identified as events with this threshold. Reported width of SEs is about 10‐fold greater than the width of slow events, again suggesting these are fundamentally different events. Second, how do these two event types relate to action potential firing? The mSEs detected with GCaMP6s in brain slices were associated with bursts of >3 action potentials although this relationship was not quantified.^13^, ^24^ KNDy neurons exhibit long‐term changes in firing rate in addition to single spikes and smaller bursts.^15^, ^68^ One postulate is that fast events arise from single spikes, slow events from bursts and the SE observed in vivo from more pronounced longer‐term changes in firing rate. Third, does senktide affect coordination of calcium events among cells? This will require longer recordings for a proper assessment. Fourth, what biological processes beyond action potential firing contribute to calcium events? Activation of Gq‐coupled receptors was mentioned above, but other aspects of cellular calcium homeostasis may also be altered, including release from and sequestration into organelles. Synchronized calcium waves occur in glia, which lack the ability to fire action potentials. This suggests confining the interpretation of calcium events to a read‐out of action potential firing may limit understanding of KNDy neuron physiology.

Finally, are there other technical differences that could account for differences in calcium event dynamics observed in this cell type? One aspect of note is that the glucose concentration in many of the prior brain slice studies was 30 mM, nearly a half log unit higher than the 10 mM used in the present work. How this difference might alter calcium handling remains to be determined. Another difference might be in limitations arising from the dynamic range of GCaMP6f. In the present study, the event detection software was designed to work under conditions of a rising baseline fluorescence as is observed with senktide treatment. The increase in frequency of fast and slow events and marked increase in amplitude of slow events during senktide treatment suggest this limitation is minimal. Location of the cells imaged could also affect interpretation, but we observed no differences based on the location of the slice with regard to the rostral‐caudal extent of the arcuate nucleus.

The present work confirms several aspects of KNDy neuron biology, as well as opening new venues for further investigation. How calcium dynamics within this population contribute to the generation of an episodic neuroendocrine signal, and how steroid feedback modifies the mechanisms of calcium handling that contribute to frequency modulation are critical areas for further defining the GnRH pulse generator.

## AUTHOR CONTRIBUTIONS

**R. Anthony DeFazio:** Conceptualization; investigation; writing – original draft; methodology; validation; visualization; writing – review and editing; software; formal analysis; data curation. **Christian R. Burgess:** Writing – review and editing; formal analysis; methodology; validation. **Laura Burger:** Investigation; methodology; writing – review and editing. **Amanda G. Gibson:** Validation; software; formal analysis; writing – review and editing. **Suzanne M. Moenter:** Conceptualization; funding acquisition; writing – original draft; methodology; validation; visualization; writing – review and editing; formal analysis; project administration; data curation; supervision; resources.

## FUNDING INFORMATION

This study was supported by the National Institute of Health/Eunice Kennedy Shriver National Institute of Child Health and Human Development (R37HD034860) to SMM.

## CONFLICT OF INTEREST STATEMENT

The authors declare no conflicts of interest.

## Supporting information


**Figure S1.** Sex, gonadal status and senktide alter calcium events in KNDy neurons, pensub ∆*F*/*F*0 analysis at the ROI level. (A) Frequency of fast (left) and slow (right) events. (B) Amplitude of fast (left) and slow (right) events. (C) Width of fast (left) and slow (right) events. For frequency, small dots are # events/duration for each ROI, large dots are mean of individual ROIs in that slice (color matched) and group mean ± SEM is shown in black. Amplitude and width, small dots are the median of events in that ROI, slice and group mean data are calculated as for frequency. Pairwise comparisons of baseline versus senktide for fast event frequency for each animal model shown on the graph. All other statistics and post hoc comparisons are in Tables S1–S4.

**Table S1.** LMM analysis of fast events detected on penumbra‐subtracted ∆*F*/*F*0 data. Bolded *p*‐values indicate the highest‐order significant interactions that were further analyzed with post hoc comparisons (Table S2).
**Table S2.** Post hoc comparison pairs for significant interactions (from Table S1) for fast events from penumbra‐subtracted ∆*F*/*F*0 analysis.
**Table S3.** LMM analysis of slow events detected on penumbra‐subtracted ∆*F*/*F*0 data. Bolded *p*‐values indicate the highest‐order significant interactions that were further analyzed with post hoc comparisons (Table S4).
**Table S4.** Post hoc comparison pairs for significant interactions (from Table S3) for slow events from penumbra‐subtracted ∆*F*/*F*0 analysis.

## Data Availability

The data that support the findings of this study are available from the corresponding author upon reasonable request.
