# Supplementary material for: Arcuate kisspeptin neurons display two types of calcium events: Regulation by sex, gonads, and senktide
Source: J Neuroendocrinol. 2026 Oct 27;38(11):e70298. doi: 10.1111/jne.70298 (PMC13711095; doi:10.1111/jne.70298)
Supplement: Supplementary file 1 [file JNE-38-e70298-s001.pdf]

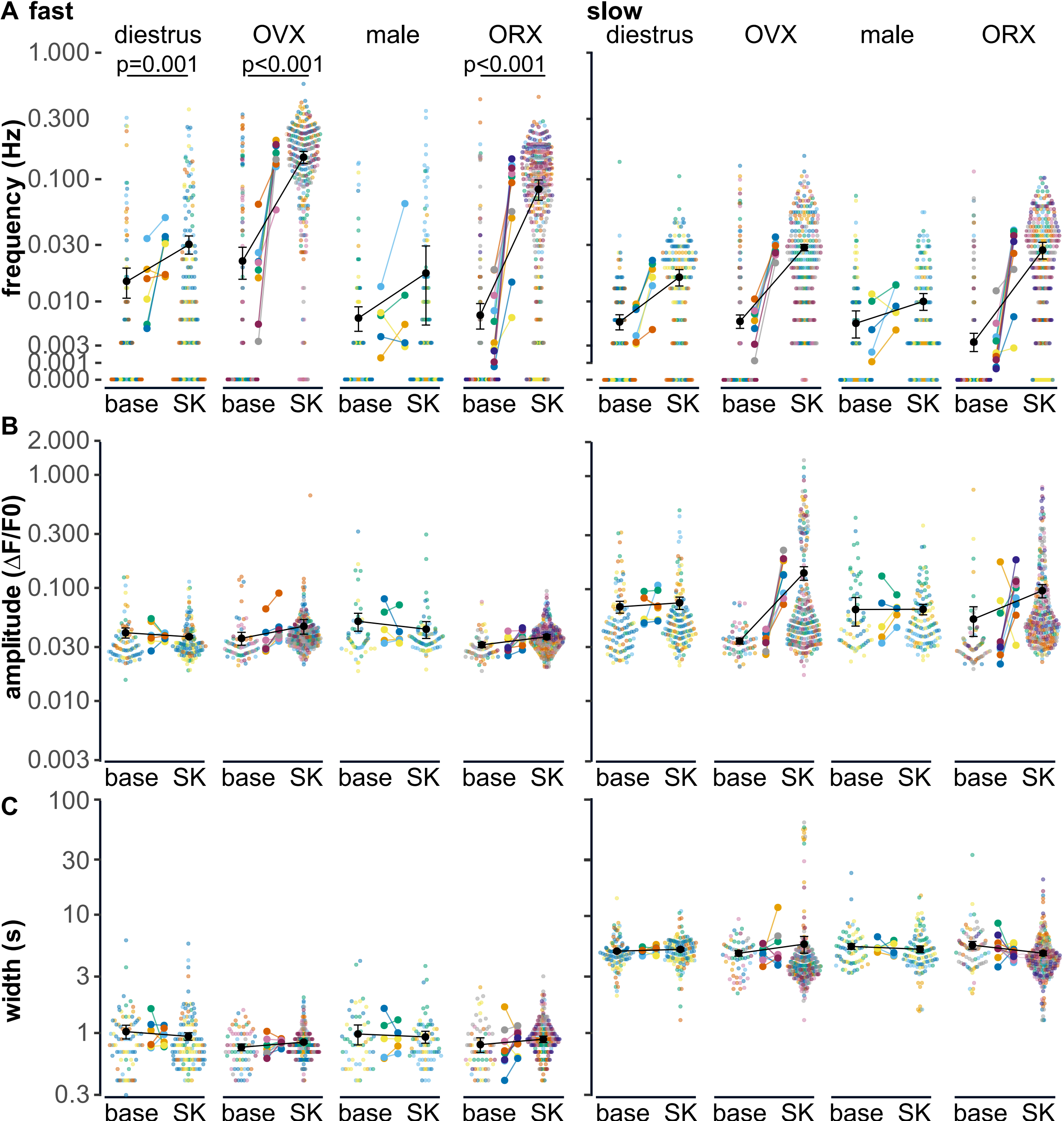

A fast
slow
diestrus
OVX
male
ORX
p=0.001
p<0.001
p<0.001
frequency (Hz)
1.000
0.300
0.100
0.030
0.010
0.003
0.001
0.000
base
SK
B
amplitude (ΔF/F0)
2.000
1.000
0.300
0.100
0.030
0.010
0.003
C
width (s)
100
30
10
3
1
0.3
