# Supplementary material for: Arcuate kisspeptin neurons display two types of calcium events: Regulation by sex, gonads, and senktide
Source: J Neuroendocrinol. 2026 Oct 27;38(11):e70298. doi: 10.1111/jne.70298 (PMC13711095; doi:10.1111/jne.70298)
Supplement: Supplementary file 2 [file JNE-38-e70298-s002.docx]

**Supplementary Table 1.** LMM analysis of fast events detected on penumbra-subtracted ∆F/F0 data. Bolded p-values indicate the highest-order significant interactions that were further analyzed with *post hoc* comparisons (Supplementary Table 2).

|  | frequency (Hz) | | | amplitude (ΔF/F0) | | | width (s) | | |
| --- | --- | --- | --- | --- | --- | --- | --- | --- | --- |
| variable | F | df | p | F | df | p | F | df | p |
| sex | 8.93 | 1,13.6 | 0.010 | 0.23 | 1,14.5 | 0.639 | 0.49 | 1,14.7 | 0.493 |
| gonad | 32.45 | 1,13.6 | <0.001 | 0.24 | 1,14.5 | 0.629 | 0.90 | 1,14.7 | 0.357 |
| senktide | 723.08 | 1,1819 | <0.001 | 8.92 | 1,999.1 | 0.003 | 3.33 | 1,1001 | 0.068 |
| sex * gonad | 1.27 | 1,13.6 | 0.279 | 3.05 | 1,14.5 | 0.102 | 0.01 | 1,14.7 | 0.928 |
| sex * SK | 25.93 | 1,1819 | <0.001 | 1.06 | 1,999.1 | 0.303 | 0.35 | 1,1001 | 0.557 |
| gonad * SK | 323.55 | 1,1819 | <0.001 | 10.15 | 1,999.1 | **0.001** | 3.13 | 1,1001 | 0.077 |
| sex * gonad * SK | 7.10 | 1,1819 | **0.008** | 0.06 | 1,999.1 | 0.811 | 0.02 | 1,1001 | 0.879 |

**Supplementary Table 2** *Post hoc* comparison pairs for significant interactions (from Supplementary Table 1) for fast events from penumbra-subtracted ∆F/F0 analysis.

| outcome | group level | contrast | estimate | 95% CI | SEM | df | t | p |
| --- | --- | --- | --- | --- | --- | --- | --- | --- |
| frequency  (sex * gonad * senktide) | intact baseline | female - male | 0.0019 | [-0.0076, 0.0114] | 0.0029 | 17.5 | 0.66 | >0.999 |
|  | GDX baseline | female - male | 0.0028 | [-0.0065, 0.0120] | 0.0024 | 8.4 | 1.17 | >0.999 |
|  | intact senktide | female - male | 0.0097 | [-0.0103, 0.0298] | 0.0061 | 17.5 | 1.59 | 0.644 |
|  | **GDX senktide** | **female - male** | 0.0754 | [0.0154, 0.1353] | 0.0154 | 8.4 | 4.90 | **0.008** |
|  | female baseline | intact - GDX | -0.0004 | [-0.0109, 0.0100] | 0.0032 | 17.5 | -0.13 | >0.999 |
|  | male baseline | intact - GDX | 0.0004 | [-0.0072, 0.0081] | 0.0020 | 8.4 | 0.23 | >0.999 |
|  | **female senktide** | **intact - GDX** | -0.1225 | [-0.1681, -0.0769] | 0.0138 | 17.5 | -8.84 | **<0.001** |
|  | **male senktide** | **intact - GDX** | -0.0568 | [-0.0921, -0.0214] | 0.0091 | 8.4 | -6.26 | **0.002** |
|  | **intact female** | **baseline - senktide** | -0.0122 | [-0.0236, -0.0007] | 0.0035 | 17.5 | -3.50 | **0.019** |
|  | intact male | baseline - senktide | -0.0043 | [-0.0116, 0.0029] | 0.0023 | 22.0 | -1.92 | 0.404 |
|  | **GDX female** | **baseline - senktide** | -0.1342 | [-0.1705, -0.0978] | 0.0112 | 19.4 | -11.98 | **<0.001** |
|  | **GDX male** | **baseline - senktide** | -0.0616 | [-0.0911, -0.0321] | 0.0076 | 8.4 | -8.14 | **<0.001** |
| amplitude  (gonad * senktide) | baseline | intact - GDX | 0.0067 | [-0.0060, 0.0193] | 0.0046 | 20.8 | 1.44 | 0.493 |
|  | senktide | intact - GDX | -0.0028 | [-0.0161, 0.0106] | 0.0046 | 12.9 | -0.60 | >0.999 |
|  | intact | baseline - senktide | 0.0003 | [-0.0062, 0.0068] | 0.0023 | 17.7 | 0.13 | >0.999 |
|  | **GDX** | **baseline - senktide** | -0.0091 | [-0.0146, -0.0036] | 0.0019 | 12.9 | -4.83 | **0.001** |

**Supplementary Table 3.** LMM analysis of slow events detected on penumbra-subtracted ∆F/F0 data. Bolded p-values indicate the highest-order significant interactions that were further analyzed with *post hoc* comparisons (Supplementary Table 4).

|  | frequency (Hz) | | | | amplitude (ΔF/F0) | | | width (s) | | |
| --- | --- | --- | --- | --- | --- | --- | --- | --- | --- | --- |
| variable | F | df | p | F | | df | p | F | df | p |
| sex | 0.95 | 1,13.7 | 0.347 | 0.01 | | 1,15.4 | 0.942 | 1.26 | 1,16.7 | 0.278 |
| gonad | 2.23 | 1,13.7 | 0.158 | 1.09 | | 1,15.4 | 0.312 | 2.56 | 1,16.7 | 0.128 |
| senktide (SK) | 703.29 | 1,1819 | <0.001 | 49.64 | | 1,1118 | <0.001 | 4.37 | 1,1117 | 0.037 |
| sex * gonad | 0.06 | 1,13.7 | 0.810 | 0.23 | | 1, 15.4 | 0.636 | 0.40 | 1,16.7 | 0.536 |
| sex * SK | 3.58 | 1,1819 | 0.058 | 0.58 | | 1,1118 | 0.445 | 3.94 | 1,1117 | **0.047** |
| gonad * SK | 141.42 | 1,1819 | **<0.001** | 37.29 | | 1,1118 | **<0.001** | 4.05 | 1,1117 | **0.044** |
| sex * gonad * SK | 2.14 | 1,1819 | 0.144 | 0.02 | | 1,1118 | 0.899 | 0.12 | 1,1117 | 0.729 |

**Supplementary Table 4.** *Post hoc* comparison pairs for significant interactions (from Supplementary Table 3) for slow events from penumbra-subtracted ∆F/F0 analysis.

| outcome | group level | contrast | estimate | 95% CI | SEM | df | t | p |
| --- | --- | --- | --- | --- | --- | --- | --- | --- |
| frequency  (gonad * senktide) | baseline | intact - GDX | 0.0018 | [-0.0011, 0.0046] | 0.0010 | 13.5 | 1.78 | 0.098 |
|  | **senktide** | **intact - GDX** | -0.0134 | [-0.0214, -0.0054] | 0.0028 | 13.5 | -4.85 | **<0.001** |
|  | **intact** | **baseline - senktide** | -0.0069 | [-0.0099, -0.0039] | 0.0011 | 18.4 | -6.43 | **<0.001** |
|  | **GDX** | **baseline - senktide** | -0.0221 | [-0.0274, -0.0168] | 0.0018 | 13.5 | -12.07 | **<0.001** |
| amplitude  (gonad * senktide) | **baseline** | **intact - GDX** | 0.0310 | [0.0064, 0.0556] | 0.0093 | 31.6 | 3.34 | **0.007** |
|  | senktide | intact - GDX | -0.0239 | [-0.0554, 0.0075] | 0.0108 | 12.5 | -2.22 | 0.092 |
|  | intact | baseline - senktide | -0.0046 | [-0.0241, 0.0148] | 0.0071 | 20.3 | -0.65 | 0.521 |
|  | **GDX** | **baseline - senktide** | -0.0595 | [-0.0784, -0.0407] | 0.0065 | 12.5 | -9.21 | **<0.001** |
| width  (sex * senktide) | baseline | female - male | -0.5344 | [-1.3017, 0.2328] | 0.2969 | 54.0 | -1.80 | 0.232 |
|  | senktide | female - male | 0.0371 | [-0.5581, 0.6323] | 0.2139 | 17.6 | 0.17 | >0.999 |
|  | female | baseline - senktide | 0.0144 | [-0.5009, 0.5296] | 0.1872 | 19.4 | 0.08 | >0.999 |
|  | male | baseline - senktide | 0.5859 | [-0.0141, 1.1859] | 0.2156 | 17.6 | 2.72 | 0.057 |
| width  (gonad * senktide) | baseline | intact - GDX | 0.0623 | [-0.7052, 0.8299] | 0.2955 | 46.8 | 0.21 | >0.999 |
|  | senktide | intact - GDX | 0.6053 | [-0.0382, 1.2488] | 0.2188 | 11.9 | 2.77 | 0.060 |
|  | intact | baseline - senktide | 0.0115 | [-0.5536, 0.5766] | 0.2094 | 24.1 | 0.06 | >0.999 |
|  | GDX | baseline - senktide | 0.5545 | [-0.0193, 1.1283] | 0.1951 | 11.9 | 2.84 | 0.060 |
